# Neurotrophic Properties of C-Terminal Domain of the Heavy Chain of Tetanus Toxin on Motor Neuron Disease

**DOI:** 10.3390/toxins12100666

**Published:** 2020-10-21

**Authors:** Mireia Herrando-Grabulosa, Caty Casas, Kevin Talbot, José Aguilera

**Affiliations:** 1 Institute of Neurosciences, Department of Biochemistry and Molecular Biology, Universitat Autònoma de Barcelona, 08193 Cerdanyola del Vallès, Spain; Jose.Aguilera@uab.cat; 2 Centro de Investigación Biomédica en Red sobre Enfermedades Neurodegenerativas (CIBERNED), 28031 Madrid, Spain; 3 Institute of Neurosciences, Department of Cell Biology, Physiology and Immunology, Universitat Autònoma de Barcelona, 08193 Cerdanyola del Vallès, Spain; Caty.Casas@uab.cat; 4 Nuffield Department of Clinical Neurosciences, University of Oxford, Oxford OX3 9DU, UK; kevin.talbot@ndcn.ox.ac.uk

**Keywords:** carboxyl-terminal domain of the heavy chain of tetanus toxin, neuroprotection, excitotoxicity, spinal muscular atrophy, amyotrophic lateral sclerosis

## Abstract

The carboxyl-terminal domain of the heavy chain of tetanus toxin (Hc-TeTx) exerts a neuroprotective effect in neurodegenerative diseases via the activation of signaling pathways related to neurotrophins, and also through inhibiting apoptotic cell death. Here, we demonstrate that Hc-TeTx preserves motoneurons from chronic excitotoxicity in an in vitro model of amyotrophic lateral sclerosis. Furthermore, we found that PI3-K/Akt pathway, but not p21ras/MAPK pathway, is involved in their beneficial effects under chronic excitotoxicity. Moreover, we corroborate the capacity of the Hc-TeTx to be transported retrogradely into the spinal motor neurons and also its capacity to bind to the motoneuron-like cell line NSC-34. These findings suggest a possible therapeutic tool to improve motoneuron preservation in neurodegenerative diseases such as amyotrophic lateral sclerosis.

## 1. Introduction

Motor neuron diseases (MNDs) are a group of neurodegenerative diseases which, despite having different etiologies and clinical patterns, demonstrate that spinal motoneurons (MNs) are selectively vulnerable. The most common form of MND in adults, amyotrophic lateral sclerosis (ALS), is characterized by combined degeneration of upper and lower MNs, leading to progressive weakness and death from respiratory failure a median of 30 months from symptom onset [1]. Most ALS cases are apparently sporadic (sALS), though about 10% show familial inheritance (fALS), and specific disease-determining mutations can be identified in up to 15% cases overall, with more than 50 genes involved. ALS is currently incurable, with available therapies such as riluzole and edaravone only having a trivial impact on disease progression [2,3]. Precision medicine based on approaches to therapy in ALS is urgently needed [4].

The carboxyl-terminal domain of the heavy chain of tetanus toxin (Hc-TeTx) is a non-toxic fragment that binds to the cell membrane [5,6]. Several studies have demonstrated that Hc-TeTx is able to preferentially bind to MNs [7,8,9,10,11], acting through lipid raft microdomains [12], through gangliosides [13,14] and glycoprotein acceptors [15,16]. However, it is still unclear which specific protein receptor mediates binding, although it is known that Hc-TeTx shares trafficking vesicles with some neurotrophins and their receptors in being transported retrogradely to the central nervous system [17]. Moreover, other functions have been described for the non-toxic domain of tetanus toxin. Hc-TeTx can rescue cerebellar granule neurons from apoptotic cell death caused by potassium deprivation and MPP^+^ toxicity [18,19], preserves MNs from acute excitotoxic damage [11], and is an effective neuroprotective agent in several animal models of neurodegeneration [20,21], including a mouse model of ALS [22,23,24]. Among the molecular mechanisms proposed for Hc-TeTx neuroprotection are the p21ras/MAPK, PI3-K/Akt, and PLCγ/PKC signaling pathways which are downstream of activation of tyrosine kinase receptors (Trks; tropomyosin-related kinases) [11,18,25,26]. However, further experiments are needed to clarify the molecular mechanisms underlying the neuroprotective effects of Hc-TeTx. Here, we wanted to demonstrate the neuroprotective mechanisms in a model of chronic excitotoxicity.

## 2. Results

### 2.1. Purification and Fluorescence Labeling of Recombinant Protein Hc-TeTx

**Figure 1:**
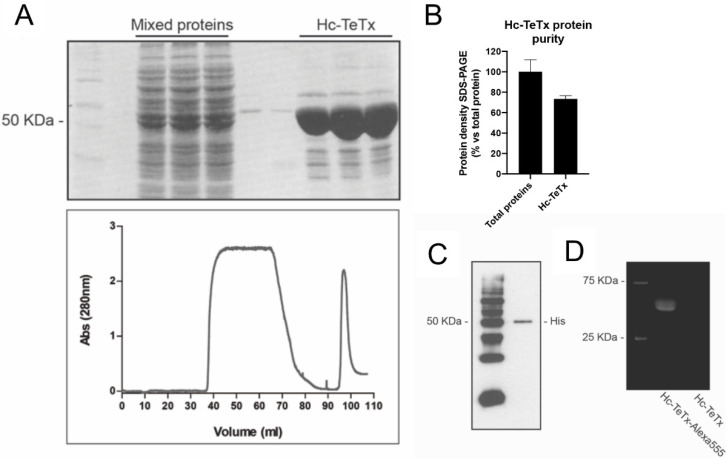
Purification and fluorescence labeling methodology of the carboxyl-terminal domain of the heavy chain of tetanus toxin (Hc-TeTx) protein. (**A**) Elution profile of the Hc-TeTx protein. Electrophoresis SDS-PAGE stained with comassie blue and fast protein liquid chromatography (FPLC) chromatogram. (**B**) Densometric quantification of the eluted lanes corresponding to Hc-TeTx fractions. (**C**) Detection of the Hc-TeTx protein purified using the Western blot technique with the anti-histidine antibody. (**D**) Labeling detection of the Hc-TeTx protein conjugated to the Alexa Fluor^®^555 fluorochrome. Histogram and electrophoretic gel representing more than 30 purification processes carried out.

In order to obtain a high-quality purification and fluorescence labeling of the recombinant protein Hc-TeTx preparation for further therapeutic treatments and retroaxonal transport analysis, we optimized the purification process of Hc-TeTx by using an FPLC system and cobalt-agarose columns. We determined the eluted protein profile by measuring the absorbance at 280 nm (Figure 1A). The chromatogram shows a wider peak that represents mixed proteins eluted, and the narrowest peak represents the elution of the Hc-TeTx. The different fractions obtained during the process of purification of the Hc-TeTx protein were treated under reducing conditions and resolved in an electrophoretic gel by loading the same volume of each sample (15 μL). A large number of proteins were observed in the wash fractions, whereas in the fractions corresponding to the narrowest peak, the presence of a protein corresponding to a molecular weight of about 50 kDa was identified, such as Hc-TeTx protein. The Hc-TeTx purity of the selected fractions represents around 70% (Figure 1B). Immunoblot with the anti-histidine antibody allowed detecting the histidine tail of the C-terminal end of the Hc-TeTx protein at 50 kDa (Figure 1C). In order to visualize Hc-TeTx by fluorescence, protein was attached to the small molecule, Alexa Fluor^®^555. Labeled as Hc-TeTx-Alexa^555^, protein could also be detected at 50 kDa under ultraviolet light (Figure 1D). Dual Color molecular weight markers indicated as M allow detecting a band at 75 kDa and a band at 25 kDa under UV. Overall, these findings show that the recombinant protein Hc-TeTx is produced at high purity and is specifically labeled by fluorescence.

### 2.2. Membrane Motoneuron Binding and Retrograde Transport of Recombinant Hc-TeTx Protein

**Figure 2:**
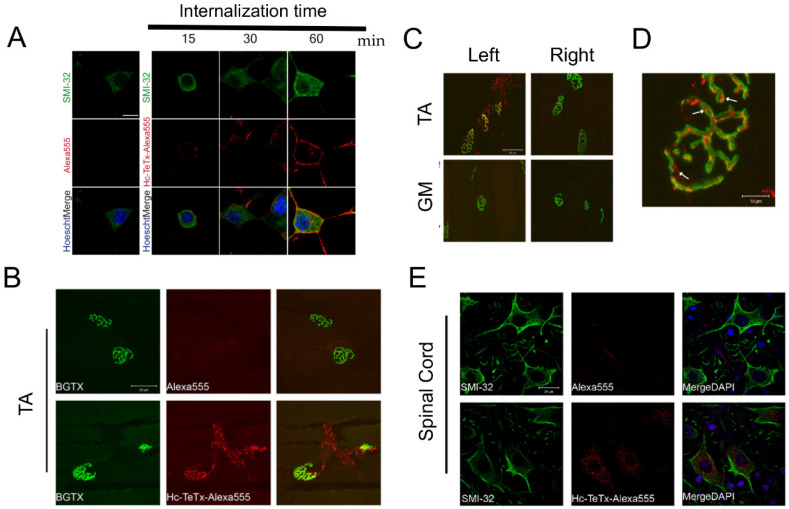
Internalization and retrograde transport of Hc-TeTx-Alexa^555^ protein on motoneurons. (**A**) Time-course of Hc-TeTx-Alexa^555^ internalization in the NSC-34 cells, a motoneuron-like cell line. Controls performed by adding the same amount of fluorochrome did not show fluorescence at the indicated times. The bar represents 10 μm. (**B**) The Hc-TeTx-Alexa^555^ (red) protein is detected in motor nerve endings identified with α-bungarotoxin (BGTX) (green) 24 h after a single intramuscular injection into the tibialis anterior (TA) muscle. The injection with the same amount of fluorocrom (Alexa^555^) does not emit fluorescence in the neuromuscular junction (NMJ). (**C**) The Hc-TeTx-Alexa^555^ protein can be detected in the injected muscle but does not spread to other muscles such as gastrocnemius muscles (GM) of the same leg (Left leg) or in the TA muscles and GM of the right leg, where there is no presence of Hc-TeTx-Alexa^555^. (**D**) At 24 h the Hc-TeTx-Alexa^555^ protein is located inside the NMJ without colocalizing with AChR receptors. The arrows indicate the formation of clusters inside the NMJ and presynaptic endings. Representative images of 3 independent experiments by condition. (**E**) Visualization of the Hc-TeTx-Alexa^555^ protein in the spinal cord at 24 h after intramuscular injection. The Hc-TeTx-Alexa^555^ protein (red) was visualized inside the soma of the motor neurons (green), which were marked with anti-SMI-32. The markup of Hc-TeTx-Alexa^555^ followed a vesicular pattern. The control injection with the same amount of fluorocrom (Alexa Fluor^®555^) did not emit fluorescence in the spinal cord.

In order to assess the capacity of the recombinant Hc-TeTx protein to be attached to MNs, we first characterized the ability of the protein to bind to a motor neuron-like cell line, NSC34. We demonstrated that upon addition of labeled Hc-TeTx to the culture, the protein was localized around the NSC-34 cells, suggesting that the incorporation of the fluorescence dye did not modify its capacity to binds to MNs. Moreover, slight labeling can be seen in the soma of NSC-34 cells at 60 min which may be due to the internalization of Hc-TeTx. However, further experiments with specific antibodies against TeTx are needed to confirm this result (Figure 2A). Interestingly, binding to the cell membranes occurs in the first 15 min after addition to the culture media. These results suggest that Hc-TeTx-Alexa^555^ maintain its properties and it can be attached and may be internalized into MNs.

In order to assess the potential for a beneficial role of recombinant Hc-TeTx proteins in MND in vivo models, we wanted to analyze the capacity of our fluorescently labeled purified Hc-TeTx protein to be transported retrogradely into the central nervous system. Hc-TeTx were administered by focal injection into the right tibialis muscle (TA) of wild type mice at 50 ng/Kg to ensure enough protein would be transported into the ventral horn MN soma. At 24 h post-injection, Hc-TeTx can be localized at the neuromuscular junction (NMJ) of the right TA muscle, co-labeled with bungarotoxin (Figure 2B). Moreover, we demonstrated that recombinant protein Hc-TeTx cannot diffuse to other muscles, such as neighboring gastrocnemius (GM), or muscles more remote from the injection site, such as the left TA and GM (Figure 2C), and it is inside the NMJ (Figure 2D). At 24 h, the recombinant protein injected into the right TA muscle can be detected in the corresponding ventral horn motor neuron pool innervating TA muscle (Figure 2E). However, further experiments with specific antibodies against TeTx are needed to confirm this result. These findings suggest that the intramuscular injection of recombinant Hc-TeTx protein can be internalized in the NMJ and transported retrogradely to the spinal cord and is an effective method of delivery of a potentially neuroprotective agent with high affinity and specificity for spinal MNs for future studies in vivo.

### 2.3. Motor Neuron Preservation under Chronic Excitotoxicity

**Figure 3:**
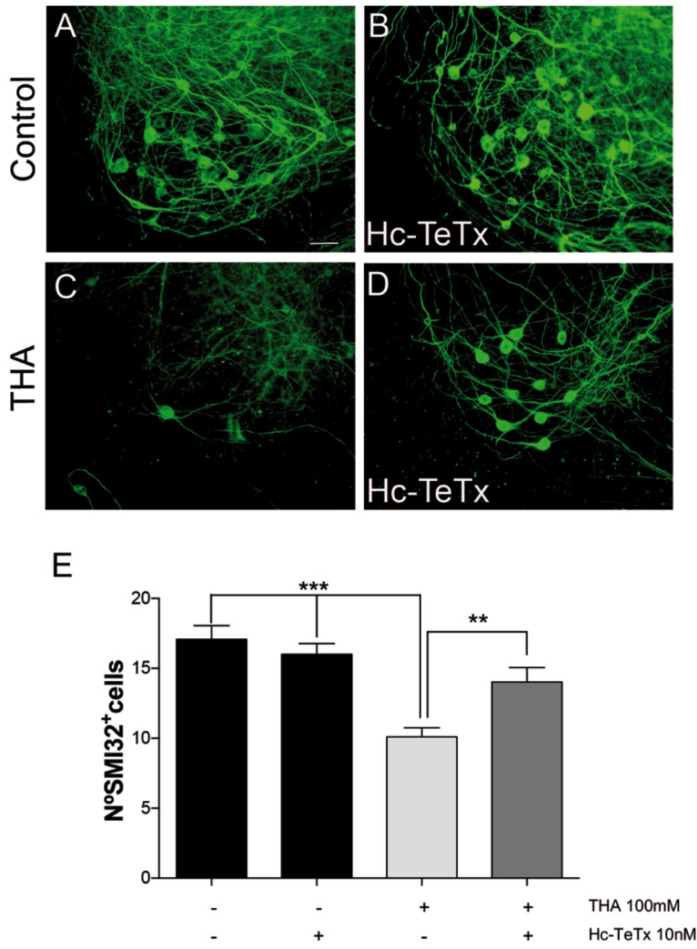
Evaluation of the neuroprotective effect of the Hc-TeTx protein against chronic excitotoxic damage caused by the addition of DL-*threo*-β-hydroxyaspartic acid (THA) (100 μM) in spinal cord organotypic cultures. Motoneurons (MNs) located in the ventral horn of the spinal cord were visualized by immunofluorescence with anti-SMI-32. The addition of THA (100 μM) at 7, 14, and 21 days in vitro (DIV) caused a loss of MNs (**C**) with respect to the control (**A**). The treatment with Hc-TeTx (10 nM) under conditions of chronic excitotoxicity allowed the viability of the MNs (**D**). There were no differences in the viability of the MNs with treatment with Hc-TeTx under controlled conditions (**B**). (**E**) Representative micrographs of the counting of positive SMI-32 cells in the ventral area of the spinal cord. The values are shown according to ± SEM with a minimum of 15 sections per treatment. On *** *p* < 0.001 and ** *p* < 0.01 with respect to THA (100 μM) according to an ANOVA analysis with the statistical test of Bonferroni. The bar represents 50 μm.

In order to assess the neuroprotective capacity of recombinant Hc-TeTx protein, we first reproduced a well-established in vitro model of progressive chronic glutamate excitotoxicity by the addition of DL-*threo*-β-hydroxyaspartic acid (THA), an inhibitor of glutamate transport, using organotypic spinal cord slice cultures. After 7 days in vitro (DIV), we administered THA, accompanied by either vehicle or recombinant Hc-TeTx protein, at 10 nM, according to previous studies [13]. THA reduces MN numbers to about 60% in comparison to the control, but the addition of Hc-TeTx allows protecting the number of MNs, reaching 80% of MN preservation. In contrast, MN counting revealed that Hc-TeTx cannot enhance the number of MNs under basal culture conditions (Figure 3). Given that chronic excitotoxicity mediated by glutamate is implicated in the pathogenesis of ALS, these results indicate that Hc-TeTx protein has the potential to act as a neuroprotective factor in ALS.

### 2.4. Signaling Cascade Induced by Hc-TeTx under Chronic Excitotoxicity

**Figure 4:**
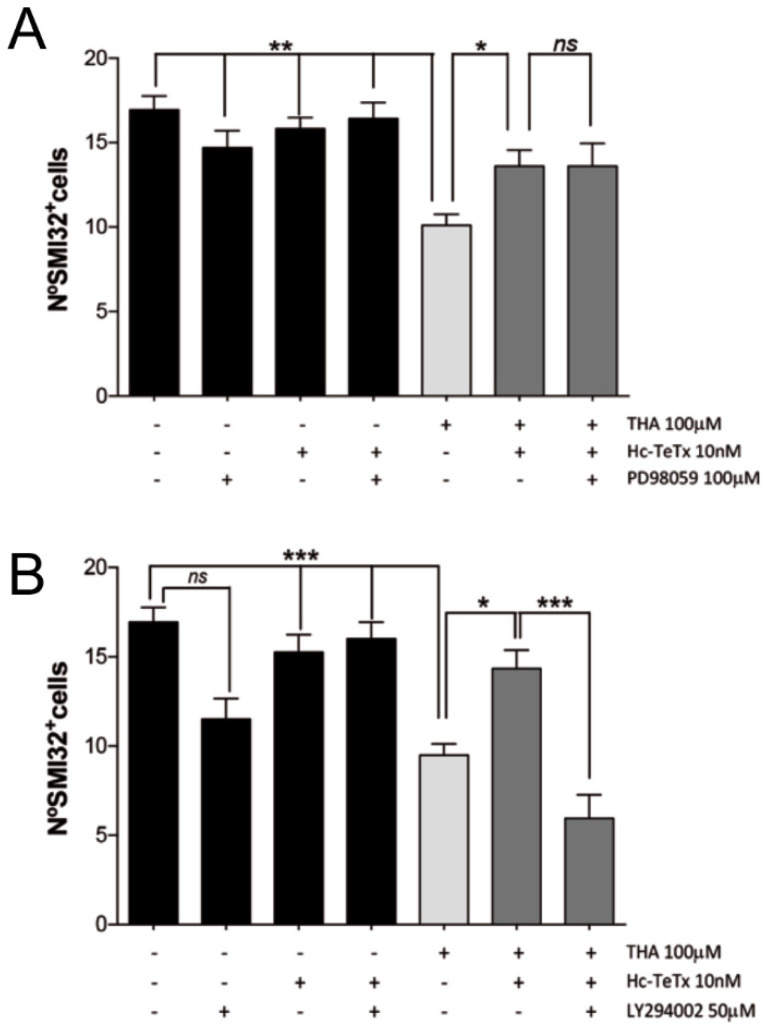
Hc-TeTx activates PI3-K/Akt signaling pathway in a chronic excitotoxicity model caused by the addition of DL-*threo*-β-hydroxyaspartic acid (THA). Organotypic cultures were treated with the glutamate transporter inhibitor (THA) at 100 μM and the Hc-TeTx protein at 10 nM. (**A**) Number of SMI-32^+^ cells countered after addition of the MEK inhibitor, PD98059 at 100μM. (**B**) Number of SMI-32^+^ cells counted after addition of the PI3-K inhibitor, LY294002 at 50μM. While the PI3-K inhibitor blocks the neuroprotective effect of Hc-TeTx protein, in the face of chronic excitotoxicity, the MEK inhibitor does not impede this ability. The values are shown according to ± SEM with a minimum of 15 sections per treatment. On *** *p* < 0.001, ** *p* < 0.01 and * *p* < 0.05 with respect to THA (100 μM) according to an ANOVA analysis with the statistical test of Bonferroni.

Previously, we demonstrated that neuroprotective effects of recombinant Hc-TeTx protein after acute excitotoxicity act via p21ras/MAPK and PI3-K/Akt signaling pathways [11]. Here, we found that the specific inhibitor for PI3-Kinases (LY294002) blocks the neuroprotective effect of Hc-TeTx under chronic excitotoxicity, but the inhibition of MEK1 (PD98059) did not block its beneficial effect (Figure 4A,B). Treatment of spinal cord cultures with both inhibitors under basal and excitotoxic conditions alone had no effect on MN survival. These findings suggest that recombinant Hc-TeTx protein exerts a neuroprotective role specifically through the PI3-K/Akt signaling pathway in an in vitro organotypic model of chronic excitotoxicity, which may have implications for targeting therapy in ALS.

## 3. Discussion

The preservation of MNs from cell death is a relevant goal in MNDs. In ALS, lack of early biomarkers of disease activity is a barrier to initiating treatment early in disease, which is likely to be the most therapeutically tractable phase. Several previous studies have suggested neuroprotective effects of Hc-TeTx in a number of neurodegenerative diseases [20,21]. Moreover, recent studies have demonstrated that Hc-TeTx promotes locomotor recovery and motor neuron survival after spinal cord injury in rats, decreasing apoptotic markers of cell death and modulating autophagy [11,27]. We have previously demonstrated that Hc-TeTx can promote the preservation of MNs under conditions of acute excitotoxic damage [11].

Here, we demonstrated that Hc-TeTx can be easily produced at high purity. Conjugation with fluorochromes facilitates easier tracking. In order to evaluate the potential neuroprotective effect of Hc-TeTx protein in ALS, we used an organotypic spinal cord model based on the inhibition of glutamate carriers by the addition of THA (100 μM) [28]. This ex vivo system is viewed as an established model of progressive excitotoxicity (slow excitotoxicity) and a very useful tool for the study of motor neuron degeneration. The origin of sporadic forms of ALS remains unclear, but it is now recognized to be a multisystemic degeneration, involving multiple pathophysiological molecular processes, including excitotoxicity [4,29]. In patients with ALS, high levels of glutamate have been detected in the cerebrospinal fluid (CSF), as well as low expression levels of the EAAT2 glutamate transporter, although whether this is the cause of MN death or a consequence remains debated [30,31,32]. Glutamate carriers play a very important role in the buffering cells due to the toxic effects of excess extracellular glutamate [33]. There is controversy around how chronically damaged motoneurons die from excitotoxicity. During the degeneration of spinal MNs, apoptotic, necrotic, and autophagic processes have been implicated, though the final common pathway of death of MNs may be mainly due to apoptosis [34,35].

The beneficial role of Hc-TeTx in an in vitro model of glutamate excitotoxicity is due specifically to the PI3-K/Akt signaling pathway. Hc-TeTx protein can also modulate autophagic pathways via PI3-K/Akt [11]. Further experiments are needed to explore the capacity of recombinant Hc-TeTx protein to preserve motoneurons and motor function in a mouse model of ALS such as SOD1G93A.

In the current study, we additionally wanted to highlight the capacity of fluorescently detecting the Hc-TeTx recombinant protein to reveal its high capacity to be internalized in MNs and to be transported retrogradely, supporting the potential of further in vivo experiments. Results from NSC34 cells reinforce the rapid capacity of Hc-TeTx to bind to MNs. Moreover, results in mice suggest that Hc-TeTx can be transported retrogradely in just 24 h.

In summary, Hc-TeTx recombinant protein preserves MNs from chronic degenerative process, specifically through the PI3-K/Akt signaling pathway. The capacity of Hc-TeTx to be transported retrogradely suggests that further work should focus on studies to elucidate the protein partners by which Hc-TeTx is transported at a faster rate than other retrotracers.

## 4. Materials and Methods

### 4.1. Recombinant Hc-TeTx Protein Purification

*Escherichia coli* BL21 cells were transformed by pQE3-Hc-TeTx-Hisx6 plasmid and cells were grown in Luria Bertani medium containing 100 µg/mL ampicillin. Protein expression was induced by the addition of 0.4 mM isopropyl β D thiogalactoside (IPTG). After 3 h, cells were pelleted by centrifugation at 4000× *g* for 20 min at 4 °C, resuspended in lysis buffer (50 mM NaH_2_PO_4_, 300 mM NaCl and 1% Triton ×100; pH 8) and sonicated on ice for six 30 s periods. The suspension was centrifuged at 30,000× *g* for 30 min at 4 °C. The clear supernatant, which contains the His-tagged protein, was purified by cobalt affinity chromatography. Mixed proteins were injected in a fast protein liquid chromatography (FPLC), which contains a cobalt-agarose resin (TALON Metal Affinity resin, Clontech Laboratories; Palo Alto, CA, USA), previously equilibrated (50 mM NaH_2_PO_4_ and 300 mM NaCl; pH 7). The proteins, without His-Tags, were eluted by washing the resin with elution buffer (50 mM NaH_2_PO_4_ and 300 mM NaCl; pH 7). Hc-TeTx contains six histidine residues and is retained in the resin forming a Co-complex. Hc-TeTx was eluted with the elution buffer (50 mM NaH_2_PO_4_, 300 mM NaCl and 150 mM Imidazole; pH 7). Fractions collected were 0.5 mL in volume. The elution process can be followed with an FPLC system that constantly measures the absorbance at 280 nm. Protein was separated by sodium dodecyl sulfate-polyacrylamide gel electrophoresis (SDS-PAGE) at 12%. Gel was stained with GelCode Blue Stain Reagent (Pierce Chemical Co.; Rockford, IL, USA) and those fractions containing purified Hc-TeTx protein were dialyzed (40 mM Na_2_HPO_4_, 10 mM NaH_2_PO_4_ and 150 mM NaCl; pH 7.4) overnight at 4 °C and for 2 h with new buffer. Protein concentrations were determined using the bicinchoninic acid assay (BCA, Pierce Chemical Co.; Rockford, IL, USA) and lyophilized. The Hc-TeTx was stored in aliquots at −20 °C. Alexa Fluor^®^555 was attached to Hc-TeTx following the manufacturer’s protocol from Alexa Fluor^®^555 Labeling Kit (Invitrogen; Carlsbad, CA, USA) and stored at −20 °C.

### 4.2. Hc-TeTx Retrogade Transport

Single intramuscular injection of Hc-TeTx-Alexa^555^ at 50 ng/kg was performed into the right tibialis anterior muscle in wild-type (WT) adult mice to assess the capacity of the conjugated protein to be retrogradely transported. After 24 h, right and left tibialis and gastrocnemius muscles were collected and fixed in PFA 4%. Then, tissue was cryopreserved with sucrose at 4 °C.

### 4.3. NSC-34 Cell Culture

NSC-34 cells were grown in coated poly-D-lysine 24-well plates with 1 mL Glutamax with 10% (*v*/*v*) FBS. Cells were plated at 2.5 × 10^5^ cells/well for experiments. After 48 h, the medium was replaced with Dubelcco Modified Eagle Medium (DMEM) plus 10% (*v*/*v*) FBS.

### 4.4. Spinal Cord Organotypic Culture

Organotypic spinal cord cultures were prepared from lumbar spinal cords of 8-day-old Sprague-Dawley rat pups (P8), as previously described [13,29,30]. After euthanasia, the spinal cord was collected under sterile conditions and placed in ice-cold high glucose-containing (6.4 mg/mL) Gey’s Balanced Salt Solution (GBSS) (Sigma-Aldrich Corp.; St. Louis, MO, USA). Meninges and roots were removed, and the spinal cord was transversely sectioned in 350 µm slices with a McIlwain Tissue Chopper (The Mickle Laboratory Engineering Co.; Surrey, UK). Five sections were carefully transferred on Millicell-CM porous membranes (0.4 µm) (Millipore; Billerica, MA, USA) into a 6-well plate containing 1 m: of incubation medium (50% minimal essential medium (MEM), 25 mM Hepes, 25% heat-inactivated horse serum, 2 mM glutamine and 25% Hank’s Balanced Salt Solution (HBSS) supplemented with 25.6 mg/mL glucose; pH 7.2). Cultures were incubated at 37 °C in a 5% CO_2_/95% air humidified environment. Cultures were left to stabilize for one week, and after this, the medium was changed twice per week until 28 days in vitro (DIV). Under these conditions, the MN population reaches a steady number from one to four weeks, as previously described [30]. All procedures involving animals were approved by the Ethics Committee of Universitat Autònoma de Barcelona and followed the European Communities Council Directive 86/609/EEC. After 7 days of the explant culture, chronic glutamate excitotoxicity will be induced by DL-*threo*-β-hydroxyaspartic acid (THA) at 100 µM, 1 to 4 weeks, a potent glutamate transporter inhibitor, which is known to produce a dose-dependent sustained elevation of glutamate levels, causing degeneration of motor neurons [28]. The neuroprotective effect of Hc-TeTx at 10 nM will be assessed by its co-addition to the culture with THA and renewal of each medium change.

### 4.5. Motor Neuron Counting

We identified MNs in the slices by immunostaining with SMI-32 antibody and according to their localization in the ventral horn of lumbar sections. All thoracic and sacral sections were excluded. We blindly counted MNs meeting these criteria in each spinal cord section. To count SMI-32 positive cells in the ventral horn of organotypic spinal cord slices, we used Z-stack confocal series.

### 4.6. Immunofluorescence

Spinal cord slices were fixed with 4% paraformaldehyde at room temperature for 1 h. Slices were then washed twice with phosphate-buffered saline (PBS) for 15 min, blocked with 5% normal horse serum and 0.2% Triton-X100 in PBS, and incubated overnight at 4 °C with antibody against mouse anti-neurofilament heavy-chain (NF-H) (SMI-32, 1:1000) (Sternberger Monoclonals Inc.; Baltimore, MD, USA). Cultures were then thoroughly washed in PBS with 0.2% Tween-20 (PBS-T) and incubated with appropriate secondary antibody Alexa Fluor^®^488 goat anti-mouse IgG (1:1000) diluted in blocking buffer for 1 h at room temperature. Then, slices were washed two times with PBS-T, incubated for 20 min with DAPI (4′,6-Diamidino-2-phenylindole), diluted in PBS, and washed several times. Finally, slices were mounted in Superfrost^®^Plus slides (Thermo Fisher Scientific; Waltham, MA, USA) with Fluoromount-G mounting medium (SouthernBiotech; Birmingham, AL, USA) and fluorescence was visualized under an epifluorescence microscope (Nikon Eclipse 90i, Nikon Instruments Inc.; Melville, NY, USA) or a confocal microscope (Leica TSC SP5, Leica Microsystems; Deerfield, IL, USA). A minimum of 15 sections were used for MN counting for each experimental condition.

### 4.7. Statistical Analysis

Statistical significance was determined by one-way ANOVA, followed by Bonferroni’s post hoc test. Differences were considered to be significant if *p* < 0.05.
